# Oral health associated with quality of life of people living with HIV/AIDS in Brazil

**DOI:** 10.1186/1477-7525-12-28

**Published:** 2014-03-01

**Authors:** Gabriella Barreto Soares, Cléa Adas Saliba Garbin, Tânia Adas Saliba Rovida, Artênio José Ísper Garbin

**Affiliations:** 1 Postgraduate Programme in Preventive and Social Dentistry, Araçatuba Dental School, Estadual Paulista University, Unesp, 1193 José Bonifácio Street, Vila Mendonça, Araçatuba, São Paulo, Brazil

**Keywords:** HIV, Oral health, Quality of life, Health services

## Abstract

**Background:**

The epidemic of HIV/AIDS enters into its fourth decade and is still considered an important public health problem in developed and developing countries. The purpose is verify the oral health and other factors that influence the quality of life of people living with HIV/AIDS attending a public service reference in Brazil.

**Methods:**

The participants answered the questionnaire on socio-demographic conditions, issues related to HIV and daily habits. The quality of life was analyzed by the HIV/AIDS Targeted Quality of Life (HAT-QoL) instrument with 42 items divided into nine domains: General Activity, Sexual Activity, Confidentiality concerns, Health Concerns, Financial Concern, HIV Awareness, Satisfaction with Life Issues related to medication and Trust in the physician. The oral health data were collected by means of the DMFT index, use and need of dentures and the Community Periodontal Index, according to the criteria proposed by the World Health Organization, by a calibrated researcher. Bivariate and multiple linear regressions were performed.

**Results:**

Of the participants, 53.1% were women and had a mean age of 42 years, 53.1% had eight years or less of schooling and 20.3% were not employed. In analyzing the quality of life domain of the HAT-QoL, with a lower average there was: Financial concern (39.4), followed by Confidentiality concern (43.2), Sexual activities (55.2) and Health concerns (62. 88). There was an association between the variables: do not have link to employment (p <0.001), is brown or black (p = 0.045), alcohol consumption (p = 0.041), did not make use of antiretroviral therapy (p = 0.006), high levels of viral load (p = 0.035) and need for dentures (p = 0.025), with the worse quality of life scores.

**Conclusion:**

Socioeconomic and inadequate health conditions had a negative impact on the quality of life of people with HIV/AIDS.

## Introduction

Acquired Immune Deficiency Syndrome is considered an important public health problem in developed and developing countries. Worldwide, there are approximately 33 million people living with HIV/AIDS, and in Brazil, it is estimated that about 630 000 individuals, 15 to 49 years old live with the disease [1,2].

After more than three decades since the beginning of the HIV/AIDS epidemic, significant improvements were observed for Antiretroviral Therapy (HAART). Because of those advances HIV has moved from a terminal illness to a chronic one [3]. With this, there was an improvement in the life expectancy of these people and the quality of life (QOL) becomes an important instrument for assessing the health of these individuals [4].

In this perspective, Locker affirms that health problems can affect the quality of life, but it is not always the case [5]. The implication is that people living with chronic diseases, such as HIV, see the disease with a dimension that other problems, such as the presence of an oral condition, for example, do not always negatively influence their quality of life [6-8].

However, it is known that dental problems can impact the health of people with compromised immune systems [9,10]. These untreated illnesses can impair chewing and swallowing [11,12], directly affect nutrition and adherence of antiretroviral therapy [13], besides influencing physically, mentally and socially to the individual’s life [14,15].

Few studies have been conducted that assessed the oral health status and quality of life of people living with HIV/AIDS. The present study investigated the quality of life associated with socio-demographic conditions, HIV-related aspects, habits and the oral health of people living with HIV/AIDS attending the public service reference in Espírito Santo, Brazil.

## Methodology

### Design study

This is a quantitative exploratory cross-sectional and analytical study carried out in the public health service offered to people living with HIV/AIDS, the STD/AIDS Reference Center, which has operated since 1992 in the capital of Espírito Santo. Espírito Santo is a state located in southeastern Brazil and despite going through a major economic development, large social inequalities are observed.

Close to 1,500 people living with HIV/AIDS are accompanied by the STD/AIDS Reference Center, on this basis, the sample size (n) was calculated by a Simple Random Sample, with a total of 177 patients, with a margin of error of 7% and a confidence level of 95%. The sample selection was carried out by randomly contacting the patients, on each examination day on a dental service, during 4 consecutive months until completed contacting the 177 subjects. We included patients with a confirmed diagnosis of HIV infection over 18 years of age and who agreed to participate in the study.

### Data collection

The collection of the data was performed over four months in 2012, after a pilot study in which all the variables were tested.

The QOL data was collected by the HAT-QoL validated instrument developed by Holmes and Shea [16], originally written in English, but translated and validated in Brazil by Galvão [17]. This instrument was selected to be specific for assessing the quality of life of people living with HIV/AIDS, and has good psychometric properties, good internal consistency and evidence of construct validity. The HAT-QoL has 42 items divided into nine domains: General Activity, Sexual Activity, Confidentiality Concerns, Health Concerns, Financial Concern, HIV Awareness, Satisfaction with Life Issues related to medication and Trust in the physician. The response for all items is obtained from a Likert scale that contains: all the time, most of the time, some of the time, part of the time and not at all. In each question, it is noted that only one option best corresponds to characterize the last four weeks experienced by the patient. The scores are calculated according to the answers, and range from one to five, with one representing a worst condition and five in a better state or condition. The scores in each domain are transformed into indices weighing 0–100, and the closer the index is to 100, the better the quality of life.

The data relating to socio-demographic questions, issues related to HIV and habits were obtained through a structured questionnaire constructed specifically for this study. The socio-demographic variables include: birth, age, gender, color, marital status, education, household income and link to employment. Those related to HIV and habits are: viral load, CD4, date of HIV diagnosis, transmission mode, use of antiretroviral therapy, smoking habits, alcohol, drug use and condom use before and after diagnosis.

The oral health examination was conducted by a calibrated researcher (kappa > 0.81 for all variables collected), in the dental clinic of the study site. To perform the test we used the probe recommended by the WHO and mouth mirrors. Condition of the teeth, gums and use/need of dentures were evaluated according to the codes and criteria for epidemiological survey of the World Health Organization [18]. For the condition of the teeth, the average number of decayed, missing or obturated teeth was used (DMFT). The periodontal status was verified by the Community Periodontal Index (CPI), which evaluated the presence of bleeding, calculus and periodontal pockets. The codes were: 0- healthy, 1- bleeding observed after probing, 2- presence of calculus, 3- bag 4–5 mm, 4- bag of 6 mm or more, X-excluded sextants (less than 2 teeth); 9- no information.

The use of dentures was recorded as follows: 0- no use, 1- fixed bridge, 2- more than one fixed bridge, 3- a removable partial denture, 4- fixed denture + removable 5- total dentures, 9- no information . The need for dentures was also evaluated according to the following codes: 0- no need, 1- one element denture, 2- more than one element, 3- combination of dentures, 4- total denture, 9- no information.

### Data analysis

Descriptive analyzes were performed for the socio-demographic characteristics of the population, through the measures of central tendency (simple frequencies, mean and median) and measures of dispersion (standard deviation). The sample involved in the research set the normal distribution of probability, assessed by the Kolmogorov and Smirnov test (p = 0.100) and the hypotheses were verified with the aid of parametric statistics. The region for the rejection or not for any of these hypotheses was considered at a significance level of 0.05. The statistical analysis of variance (ANOVA) and multiple linear regression tests were conducted with elimination of the non-significant variables. The selection of independent variables for the multiple linear regression models was performed according to a stepwise model. After adjusting the multiple linear regression model, with a confidence interval of 95%, the p- values were estimated. All statistical analyzes were performed using the SPSS version 17.0.

### Ethical aspects

The study was approved by the Ethics Committee on Human Research of the Universidade Estadual Paulista, School of Dentistry of Araçatuba and performed with the understanding and written consent of each participant.

## Results

Of the study participants, more than half of the sample were women (53.1%) with a mean age of 42 years. Most were brown (61.6%) and were not in a stable relationship (48.6%). Regarding education, 53.1% had eight years or less of schooling and 7.3% could not read and write. Of the participants, 20.3% were not employed and 63.8% had a monthly income of one to two minimum wages. Regarding the tests, 78.2% had CD4 counts greater than 350 cells/mm3 and 61.6% undetectable viral load. Regarding the discovery time of HIV diagnosis, 35.0% responded that there was more than 10 years and 86.4% of patients were aged 20 to 50 years when they received the result. The transmission by heterosexual relations was 55.9% and 53.1% of the respondents said they did not know where they contracted the virus. Most made use of the HAART (77.4%). Of the patients, 77.4% were non-smokers, 67.2% did not consume alcohol and 95|.5% of the patients said they did not use illicit drugs. Regarding the use of condoms during sexual intercourse, 67.8% said they always use and 6.8% said they never use, even after the discovery the HIV.

Patients had a mean DMFT of 17.64 (standard deviation = 7.786) (2.85 decayed teeth, SD = 3.58, 9.12 missing teeth, SD = 7.95 and 5.67 obturated teeth, SD = 4.99). The majority of the HIV^+^ patients showed good periodontal status (56.6%) and 25.3% had periodontal pockets of 4 to 5 mm. Regarding the use of dentures, 35% of those surveyed used dentures. Among them, 1.69% used fixed bridges, 18% removable partial dentures, 10.7% total dentures. Regarding the need for dentures, 41.5% needed dentures in the maxilla and 62% in the mandible.

In the assessment of the QoL, the domain with the lowest average was the Financial concern (39.4), followed by the Confidentiality Concern (43.2), Sexual activities (55.2) and Health concern (62.88), and the highest averages were: Trust in physician (96.18) and Issues relating to medication (86.54).

The results of the bivariate analysis performed between the averages of the HAT-QoL domains (dependent variables) with the socio-demographic variables, HIV-related aspects, habits and oral health can be seen in Table 1.

**Table 1:** Bivariate analysis of the standard scores of the HAT-QoL domains according to the socio-demographic variables of the 177 participants of the study in Brazil, 2012

**HAT-QoL domains**									
**Variables**	**General activity**	**Sexual activity**	**Confidentiality concern**	**Health concern**	**Financial concern**	**HIV acceptance**	**Satisfaction with life**	**Questions related to medication**	**Trust in physician**
Socio-demographic variables									
Age									
18-34	81.8	65.4	35.1	55.6	48.4	67.7	81.8	84.2	92.6
35-44	76.4	59.5	41.2	61.8	30.7	70.8	77.2	88.6	97.7
45-70	71.8	45.2	49.8	68.0	42.2	83.2	77.7	86.6	96.7
p value	0.109	*0.014*	*0.017*	*0.004*	*0.021*	*0.021*	0.687	0.476	0.157
Sex									
Female	72.0	45.8	39.0	58.8	33.1	69.0	72.0	85.1	94.9
Male	80.2	65.8	47.7	67.4	46.4	81.7	85.8	88.7	97.5
p value	*0.025*	*0.001*	*0.041*	*0.003*	*0.009*	*0.009*	*0.001*	0.184	0.198
Color									
White	72.3	59.9	55.5	64.8	44.4	82.5	75.6	92.3	98.9
Black	76.3	52.8	40.2	63.5	42.1	76.9	81.8	87.2	99.1
Brown	76.6	54.7	40.9	62.1	37.0	72.2	78.1	85.1	94.4
p value	0.692	0.740	*0.026*	0.773	0.491	0.285	0.644	0.158	0.089
Marital status									
Single	80.2	54.8	45.2	62.9	38.8	73.8	80.0	88.0	97.9
Married/Consensual union	74.0	70.0	46.2	66.4	38.3	85.5	82.2	82.8	97.2
Widowed	63.0	12.8	36.5	61.1	43.7	64.7	64.9	89.8	91.6
Separated/Divorced	74.1	50.4	48.7	64.5	45.1	77.9	76.8	85.9	90.8
p value	0.114	*0.000*	0.216	0.687	0.900	0.312	0.425	0.722	0.177
Education									
Illiterate	62.6	50.6	42.6	66.1	18.3	69.9	76.0	78.8	93.6
8 or < of study	76.8	50.5	44.3	61.7	37.3	71.6	78.7	86.6	96.7
9 years or more of study	75.1	57.7	42.6	62.7	42.4	79.5	76.2	89.1	96.2
p value	0.162	0.079	0.921	0.683	*0.015*	0.325	0.663	0.335	0.834
Link to employment									
Employed	84.4	69.7	36.7	64.4	46.7	77.9	85.3	88.7	95.9
Not employed	67.9	41.8	49.2	61.4	32.5	72.2	72.2	85.0	96.3
p value	*0.000*	*0.000*	*0.002*	0.296	*0.005*	0.246	*0.002*	0.167	0.839
Personal income									
Less than 1 MS	57.0	41.6	52.5	56.1	18.0	72.2	60.5	89.5	92.1
1 to 2 MS	77.0	53.1	41.0	63.8	39.2	74.1	80.2	87.1	96.9
3 to 4 Ms	83.7	68.6	47.2	66.2	53.5	81.3	82.7	86.3	97.4
More than 4 MS	84.4	55.0	36.0	61.0	49.0	76.8	94.4	85.0	96.6
p value	*0.002*	0.146	0.684	0.557	*0.006*	0.572	*0.003*	0.931	0.123
Variables related to HIV and habits									
VL									
Undetectable	77.4	53.9	46.1	65.2	39.3	78.9	81.6	88.3	97.7
<10000	73.9	56.9	42.8	62.6	44.1	68.8	82.1	90.7	96.3
>10000	74.2	59.0	38.3	60.6	38.3	70.3	70.0	79.8	91.8
p value	0.689	0.757	0.290	0.399	0.796	0.196	0.062	*0.016*	0.059
CD4									
<200	57.4	59.2	47.2	72.2	29.8	79.6	60.5	82.6	98.1
From 200 to 350	72.9	60.4	43.3	54.1	39.8	63.4	73.0	80.7	91.0
>350	76.0	55.6	43.4	63.7	39.5	75.4	78.5	86.4	96.0
p value	*0.039*	0.722	0.915	*0.006*	0.680	0.096	0.058	0.145	0.112
Years since Discovering HIV									
Less than 1 year	72.7	52.0	31.2	41.2	39.1	61.3	70.3	78.7	86.5
From 1 to 5 years	80.3	63.7	39.5	65.5	42.6	70.0	79.6	86.5	94.5
More than 5 years	78.0	58.1	42.0	66.0	40.2	77.6	84.6	89.2	96.9
p value	0.155	0.089	0.146	*0.003*	0.774	0.294	0.126	0.447	0.098
Transmission mode									
Heterosexual relations	71.4	49.3	41.7	59.6	36.0	69.8	74.9	83.3	95.1
Homosexual relations	84.9	68.5	46.6	67.0	50.6	87.7	88.1	90.2	96.7
Use of drugs	100.0	100.0	80.0	45.0	0	100.0	81.0	81.0	100.0
Blood transfusion	69.5	0	57.5	70.0	44.0	100.0	100.0	94.0	100.0
Mother to child	100.0	50.0	70.0	77.5	84.5	100.0	94.0	100.0	100.0
p value	*0.043*	*0.026*	0.370	0.146	0.065	*0.046*	0.167	0.096	0.891
From whom contracted									
Actual partner	79.1	55.5	28.3	50.8	42.8	86.1	68.1	88.5	77.8
Ex partner	57.8	26.2	45.7	57.1	27.1	64.8	61.6	84.5	92.5
Mother	100.0	50.0	70.0	77.5	84.5	100.0	94.0	100.0	100.0
Do not know	79.1	62.0	43.8	65.4	40.0	77.8	81.9	89.4	98.6
p value	*0.026*	*0.012*	0.075	0.323	0.203	0.247	0.124	0.349	*0.006*
Use of HAART									
Yes	76.3	54.7	45.4	63.3	39.7	77.5	80.8	88.4	97.8
No	74.0	57.0	35.8	61.2	38.1	66.2	70.5	81.3	90.6
p value	0.600	0.742	0.053	0.547	0.797	0.051	*0.040*	*0.028*	*0.003*
Non use of HAART									
Yes	64.0	49.4	53.7	57.1	22.0	61.4	65.6	83.0	97.9
No	80.6	55.9	43.0	65.5	46.0	82.3	85.8	90.1	97.7
p value	*0.000*	0.406	*0.048*	*0.029*	*0.000*	*0.000*	*0.000*	0.075	0.930
Smokers									
Yes	75.1	63.5	39.6	62.7	25.9	79.1	73.1	83.7	98.7
No	76.0	52.8	44.3	62.9	43.3	73.7	80.1	87.7	95.4
p value	0.978	*0.025*	0.721	0.519	0.602	0.816	0.930	0.064	0.103
Consumes alcoholic drinks									
Yes	77.7	65.3	38.6	60.4	33.2	75.4	78.5	85.1	95.8
No	74.9	50.3	45.5	64.0	42.3	74.7	78.5	87.6	96.3
p value	0.474	*0.015*	0.117	0.242	0.094	0.897	0.993	0.379	0.810
Use of illicit drugs									
Yes	71.8	50.8	55.0	43.7	26.6	61.5	55.3	68.7	89.6
No	76.0	55.4	42.6	63.7	40.0	75.6	79.6	87.6	96.4
p value	0.638	0.745	0.215	*0.004*	0.277	0.229	*0.016*	*0.003*	0.162
Used contraception before HIV									
Never	74.8	47.5	46.7	63.9	36.8	73.4	78.2	88.8	98.2
Always	74.7	64.6	45.0	77.5	46.8	75.0	68.0	85.0	91.6
Sometimes	76.4	64.6	45.0	77.5	78.5	100.0	94.0	100.0	100.0
p value	0.841	*0.000*	0.249	0.603	0.380	0.286	0.455	0.678	0.055
Oral health variables									
Caries									
≤ 3	75.5	52.9	42.0	62.5	62.5	40.6	78.6	86.8	96.6
>3	76.5	60.2	45.8	63.5	63.5	36.5	78.2	86.7	95.1
p value	0.810	0.24	0.403	0.761	0.461	0.995	0.927	0.972	0.497
Teeth loss									
≤9	76.2	58.8	40.5	60.3	39.2	74.6	79.6	86.5	95.2
>9	75.6	49.0	49.3	66.5	41.0	77.6	77.7	87.5	97.9
p value	0.861	0.106	*0.036*	*0.039*	0.076	0.544	0.671	0.725	0.191
Obturated									
≤6	77.1	57.8	47.0	64.4	41.2	76.8	80.5	86.1	97.7
>6	73.9	50.2	38.2	59.5	37.5	73.7	76.0	88.3	97.1
p value	0.398	0.212	*0.041*	0.109	0.487	0.540	0.301	0.436	0.516
DMFT index									
≤18	78.0	58.1	41.4	59.8	39.4	71.8	82.5	84.4	94.6
>18	73.6	52.2	45.1	66.0	39.3	78.2	74.4	89.3	97.7
p value	0.231	0.315	0.374	*0.033*	0.977	0.191	0.055	0.069	0.120
PCI									
≤2	41.6	33.3	50.0	50.0	33.3	44.3	47.6	79.0	100.0
>2	83.4	50.3	40.6	64.3	48.4	73.5	82.9	90.3	98.5
p value	*0.022*	0.759	0.304	0.707	0.273	0.182	0.278	0.074	0.445
Use of dentures									
Yes	76.8	44.9	45.3	68.6	43.8	80.1	81.5	91.2	99.1
No	75.4	59.2	42.4	60.6	37.6	72.9	77.3	85.1	95.0
p value	0.751	*0.026*	0.533	*0.013*	0.273	0.186	0.379	0.041	0.066
Denture need									
Yes	72.2	50.5	44.7	62.8	36.5	72.8	75.8	87.0	97.1
No	81.4	62.1	40.6	62.6	44.6	78.3	82.6	86.3	94.5
p value	*0.014*	0.053	0.338	0.927	0.121	0.276	0.116	0.817	0.211

Note: values in italic: p < 0.05.

For socio-demographic variables there was a statistically significant difference between the ages in the domains of Sexual Activity, Confidentiality concern, Health concern, Financial concern and HIV acceptance. Regarding the sex variable, it was observed that differences between groups were statistically significant in six domains of the HAT-QoL, except in the domains Questions related to medication and Trust in physician.

Regarding personal income, we found that, of the nine domains in only three were observed statistically significant differences: General Activity, Financial concern and Satisfaction with life. In the clinical variables, only the viral load was associated with the domain Questions related to medication and CD4 with General Activity and Health concern.

For the variable related to the transmission of HIV, how transmission occurred was associated with the domains General Activity, Sexual Activity and Satisfaction with life. Regarding the abandonment of treatment we observed statistically significant differences in the domains: General Activity, Confidentiality concern, Health concerns, Financial Concern, HIV acceptance and Satisfaction with life. Alcohol consumption and smoking were associated with Sexual activity. And drug use with the domains Health concerns, Satisfaction with life and Questions related to medication.

Regarding the variables of oral health condition, there were statistically significant differences: number of missing teeth with Confidentiality concern and Health concerns; filled teeth with Confidentiality concern; DMFT with Health concern, use of maxillary denture with Sexual activity, Health concern and Questions related to medication; need for denture with General Activity.

In the multiple linear regression model for the general domain activity, good periodontal status and the good levels of CD4 cells had positive effects on the QoL, while did not have link to employment and the need for dentures had negative effects. Negatively associated to the Sexual activity domain, it did not have link to employment and alcohol consumption (Table 2).

**Table 2:** Multiple linear regression for the quality of life variable for the 177 participants of the study

**Quality of life domains of the HAT-Qol**	**Explicative variables**	**B**	**p value**
**General activity (r**^**2**^ **= 0.506)**	No link to employment	-16.375	0.000
	Good periodontal status	6.025	0.000
	Good levels of CD4	9.328	0.007
	Need for dentures	-8.368	0.025
**Sexual activity (r**^**2**^ **= 0.318)**	No link to employment	-19.943	0.001
	Consumes alcoholic beverages	-12.758	0.041
**Confidentiality concerns (r**^**2**^ **= 0.311)**	Older age	7.534	0.007
	Brown or black	-5.684	0.045
**Health concerns (r**^**2**^ **= 0.437)**	Of the masculine sex	10.289	0.001
	Denture user	8.675	0.016
	Do not use illicit drugs	20.722	0.006
	Younger age	3.890	0.046
**Financial concerns (r**^**2**^ **= 0.462)**	Higher level of education	10.430	0.002
	Non smoker	19.784	0.002
	Good periodontal status	6.995	0.002
	No link to employment	-15.601	0.004
**HIV acceptance (r**^**2**^ **= 0.359)**	Good levels of CD4	11.323	0.024
	HIV diagnosed more than10 yrs yrsanos	6.927	0.018
	Higher level of education	10.529	0.002
	Good periodontal status	5.941	0.008
**Satisfaction with life (r**^**2**^ **= 0.390)**	Of the masculine sex	15.993	0.000
	Good levels of CD4	14.332	0.001
	Use of HAART	-12.813	0.016
**Questions related to medication (r**^**2**^ **= 0.334)**	Use illicit drugs	18.609	0.013
	Sexual transmission mode	1.709	0.020
	High levels of VL	-3.590	0.035
**Trust in the physician (r**^**2**^ **= 0.226)**	Non use of HAART	-7.781	0.006

*B = Regression model coefficient. r = Determination coefficient*. Brazil. 2012.

Concerning the Confidentiality domain, older age was positively associated with the best scores, while the fact that being brown or black had a negative association.

In the analysis for the Concern with health domain, being male, of younger age, using dentures and not using illicit drugs were associated with higher scores on the QoL. Concern for the financial domain was associated with the higher score variables: higher level of education, nonsmoking and good periodontal status, while not having link to employment was associated with lower scores.

In the Awareness domain about HIV, good levels of CD4 cells were found, HIV diagnosis of more than 10 years, higher level of education and good periodontal status were associated with the better scores. HAART use was negatively associated with the Satisfaction with life domain, while being male and having good levels of CD4 cells were positively associated. Issues related to variable drugs, not using illicit drugs and not having been infected through sex were positively associated, while high viral load was negatively correlated, as to the Trust the physician domain, not using the HAART was negatively associated.

## Discussion

The results of this study showed that the oral health status of the participants was worse than the national average of Brazilians [19], with a DMFT index of 17.64, with a mean of 9 missing teeth and 3 decayed teeth per person, although large associations of the oral health variables in the quality of life domains were not observed. This can be explained by the fact that oral problems, in most cases, does not cause a threat to life, being, in general, acute episodes and readily treatable. Thus, their impact on the quality of life may not be obvious and often minimized by the context of other more serious chronic conditions [20]. In addition, low socioeconomic status and low education of this population put them in disadvantage in relation to oral health problems due to lack of access to dental care, and lack of information about the prevention of oral diseases. And that’s why they do not give much importance to oral health, which also explains the low impact of oral health on quality of life [21,22].

In Brazil, few studies have reported the experience of dental caries in people living with HIV/AIDS and those that were made most were related to children. It is known that dental caries is an infectious disease and the various side effects of antiretroviral therapy may contribute to inadequate oral hygiene and diet, that being said, HIV infection appears to be a risk factor for caries [23]. This can be observed by the results of the oral health of this study, as well as others performed with people living with HIV/AIDS, where high levels of DMFT were reported. And these numbers are far from the goals proposed by the WHO for the year 2010, where the presence of 20 or more teeth in the oral cavity for 96% of the individuals with no missing teeth, at 18 years of age [24-26].

Oral diseases and HIV infection have a higher impact on disadvantaged and socially marginalized populations [27] similar to this study, where the majority of the respondents were poor and had little schooling. Another important aspect with regard to the transmission mode, which largely consisted of heterosexual relationships, is in agreement with the epidemiological profile of HIV a few years ago: heterosexuals and women, impoverishment and internalization [28,29].

The number of decayed and missing teeth and the need for dentures characterize the sample as a group in great need of dental care [30] and in Brazil, access to free dentistry treatment is still complicated, there is a long waiting list for patients with oral problems and they do not have the financial means to afford a private service, and give up dental care. In addition, the Brazilian population shows that the demand for dental care happens when the dental disease is already at an advanced stage, with irritation and pain. A direct consequence of this systematic exclusion of services, are dental extractions which, in most cases, could be avoided [31].

In the analysis of the scores of the quality of life, the results showed that the lowest averages were: Financial concern, Confidentiality concern, Sexual activities and Health concerns, results that corroborate with the findings of other studies using the HAT –QoL scale [16,17,32].

The average obtained (39.4) in the Financial concern domain probably was the low per capita income of the respondents, which hinders the survival of the individual. This may indicate that income, in spite of holding their value on living conditions and services, can influence the state of health, which further hinders their integration into the labor market [33]. People living with HIV/AIDS who have no link to employment have a poorer quality of life than those who work, this is also observed in another study [34]. Therefore, the chronicity of the disease brings new changes, including issues related to occupation, employment appears to not only have the importance of financial benefit, but is a form of social, emotional and identity inclusion as well [35].

The Confidentiality concern is a very common situation among these individuals, which happens because of the fear of being seen as having a disease still very stigmatized by society. That means they have a life of duplicity, because only a few people from their neighborhood are chosen to know about their HIV status [32]. This concern even affects the demand for health care because these patients stop going, or looking for the dentist too late, for fear of having to report that they have the HIV virus and suffer prejudice due to the unpreparedness of the professional, this makes these patients prone to having major problems related to oral health, this can be observed in the significant association of the variables of decayed and teeth loss with the Confidentiality concern domain [36].

Life with HIV/AIDS brings changes in the sexual activity of persons, and the conditions under which they find themselves due to the infection, leading to a fear of sexual relationships, which makes them avoid relationships, even though they have sexual desires [37]. This implies the involvement of the Sexual activity domain on the quality of life and the fact that there is no link to employment and alcohol consumption further strengthens a poorer quality of life.

The Health concern domain investigates the restlessness of the respondents living with the virus and its consequences, and self-assessment in relation to health, knowledge of laboratory parameters and concern with a prognosis of death situations that possibly hinder well-being [17,38]. From this perspective, in this study the year of HIV diagnosis was associated with this domain, for the reason that people with recent discovery of the infection do not accept the fact that the disease, which leads to discouragement with life and health care, and implies low scores . In the multiple linear regression, the fact of not using illicit drugs had a positive association in this field, in addition to using dentures, which demonstrates the care of individuals in their health and wellbeing.

The periodontal status of the participants of the study was good on the most part, with the Community Periodontal Index below the expected average, as well as the study by Lemos et al., who found a low frequency of periodontal disease in people living with HIV/AIDS, and the condition of systemic infection an important factor in maintaining the periodontal status. Individuals with a more advanced state of infection presented greater problems in the periodontium. This positively influences the periodontal health on the quality of life in more than one domain, as observed in the multivariate analysis [39,40].

The found results presented the socio-demographic and clinical characteristics of these individuals similar to other surveys conducted with a Brazilian population living with HIV/AIDS [17,34,35]. There are some limitations to this study, such as the voluntary participation of the respondents, which may have had more individuals concerned about the oral and general health and higher education. The research is cross-sectional, which precludes causal inferences. Due to the anonymous nature of the study, it was not possible to collect any information about those who did not answer the questionnaire completely. With regards to the quality of life instrument, though it may be validated in the Portuguese language, the educational level of the respondents may have had an influence on the responses in which the participants responded without even understanding the question.

Despite these limitations, the study shows that there are many factors that influence the quality of life of people living with HIV/AIDS, and the socioeconomic conditions and inadequate oral health negatively influence the quality of life of these individuals.

## Competing interests

The authors declare that they have no competing interests.

## Authors’ contributions

GBS, CASG and AJIG designed the study, gathered the information, performed the statistical analysis and wrote the first draft of the manuscript. TASR designed the form for data gathering and supervised the statistical analysis. All authors read and approved the final manuscript.
